# Texas State Medical Association

**Published:** 1900-03

**Authors:** A. B. Gardner

**Affiliations:** President T. S. M. A.; Bellville, Texas


### Society Notes.

### Texas State Medical Association.

BELLVILLE, TEXAS, March 15, 1900.

DEAR DOCTOR: The Texas State Medical Association will meet in the city of Waco, on Tuesday, the 24th of April, 1900, and you are earnestly requested to attend, and induce your neighbor doctors to attend also. We desire to have every regular physician in the State to co-operate with us. There is much of interest to the profession that should be considered at this meeting. The indications are that we will have one of the best meetings, both from a scientific and social standpoint, that we have ever had. Waco is a delightful city in which to meet, and I can assure all who attend a pleasant and profitable week. So throw off busy cares for a few days and come, and let us all reason with one another. You will be amply repaid for your time. Bring your wives and daughters, they too will enjoy the visit. The committee of arrangements will, in due time, notify you as to railroad rates and hotel accommodations, which I feel sure will be as reasonable as we could expect.

Yours truly,

A. B. GARDNER, M. D.,
President T. S. M. A.

